# A Closed-Form Analytical Solution for the Axisymmetric Compression of Packer Rubber Cylinders Based on the Mooney–Rivlin Model

**DOI:** 10.3390/ma19184010

**Published:** 2026-09-20

**Authors:** Jianyu Li, Peng Jia, Hang Li, Chenliang Ruan, Heming Zhu, Hongqian Liao, Xinliang Li

**Affiliations:** 1 SINOPEC Research Institute of Petroleum Engineering Co., Ltd., Beijing 102206, China; lijy96399.sripe@sinopec.com (J.L.); lihang.sripe@sinopec.com (H.L.); ruancl.sripe@sinopec.com (C.R.); zhuhm.sripe@sinopec.com (H.Z.); liaohq.sripe@sinopec.com (H.L.); lixl@tju.edu.cn (X.L.); 2 College of Pipeline and Civil Engineering, China University of Petroleum, Qingdao 266580, China

**Keywords:** compression packer, rubber cylinder, Mooney–Rivlin model, large deformation, analytical solution

## Abstract

**Highlights:**

**What are the main findings?**

- Closed-form Mooney–Rivlin solution for three-stage packer compression.
- Frictionless theory predicts average contact pressure under friction.
- Linear contact-pressure–axial-force relation identified.

**What are the implications of the main findings?**

- Enables fast packer design without nonlinear FE runs.
- Provides conservative contact pressure estimate for safety.
- Offers simple tool to determine setting force from seal pressure.

**Abstract:**

Compression packers are widely used in oil and gas well operations for zonal isolation, yet the large-deformation mechanical behavior of their rubber sealing elements lacks a closed-form analytical solution. This paper presents a complete theoretical analysis of the axisymmetric compression of an annular rubber cylinder based on the incompressible Mooney–Rivlin hyper-elastic model. The deformation process is divided into three successive stages: free expansion, casing-constrained deformation, and fully constrained deformation. Analytical expressions for the principal stretches, stress fields, and axial force are derived for each stage by integrating the radial equilibrium equation with proper treatment of the Lagrange multiplier. The frictionless analytical results are validated against axisymmetric finite element simulations, showing excellent agreement for all stress components. The applicability of the frictionless theory to frictional conditions is then examined. Results show that although friction introduces non-uniform axial deformation and end bulging, the average contact pressure on the rubber–mandrel interface agrees closely with the theoretical prediction, especially at higher axial forces (150–250 kN). A linear relationship between the average contact pressure and the axial force is confirmed, providing a simple design tool. The theoretical solution offers a computationally efficient alternative to finite element analysis for preliminary packer design and parametric studies.

## 1. Introduction

Packers are indispensable downhole tools in oil and gas development, serving as the primary means of sealing the annulus, isolating the production layer, and controlling the production (injection) of fluids [1]. They are deployed across multiple stages of a well’s lifecycle, including drilling [2], cementing [3], acidizing [4], fracturing [5] and production [6,7], to prevent fluid migration between adjacent formations and enable selective stimulation or injection operations [8]. As the oil and gas industry moves toward deeper reservoirs and harsher downhole environments, the reliability of packer sealing systems has become increasingly critical for safe and economical field development [9].

Packers can be classified into three categories based on their setting mechanism: compression packers [10], expandable packers [11], and swellable packers [12]. Among these, compression packers are the most widely used in multistage hydraulic fracturing and production operations, owing to their simple mechanical structure, reliable setting mechanism, compatibility with high differential pressures, and insensitivity to wellbore fluid composition [13].

The rubber cylinder is the core sealing component of a compression packer [14]. Extensive research has been devoted to understanding its mechanical behavior under axial compression, with contributions from both theoretical analysis and finite element (FE) simulation.

On the theoretical front, Zhang et al. [15] divided the rubber tube deformation into free deformation and constrained deformation stages, and established the deformation equations based on classical elasticity theory; the contact pressure and compression length were calculated iteratively using Hooke’s law and the geometric relations of small-strain elasticity. Dong et al. [16] derived a linear-elastic axisymmetric displacement equation from micro-element equilibrium and used the Yeoh hyper-elastic finite element method (FEM) to design an HPS-PSP packer. Jin et al. [17] derived an axisymmetric analytical solution for a packer sealing cylinder treated as a perfectly elastic material under generalized Hooke’s law. Zhang et al. [18] presented an analytical model for the compression packer, treating the rubber as homogeneous and perfectly elastic, governed by generalized Hooke’s law. Chen et al. [19] used linear-elastic thick-cylinder theory plus empirical contact formulas for seating force, leaving finite-strain hyper-elastic stress fields unclosed. He et al. [20] used Lamé thick-cylinder linear elasticity for nominal stress and Yeoh hyper-elastic FE analysis to evaluate the packer’s sealing performance.

The above theoretical studies on packer rubber cylinders are mostly limited to linear elasticity and small-strain assumptions, which cannot capture the hyper-elastic large deformation of rubber sealing elements. However, outside the packer-specific literature, the axisymmetric finite deformation of incompressible hyper-elastic tubes has been studied since the 1950s.

Wilkes [21] studied the bifurcation of a thick-walled incompressible neo-Hookean tube under axial compression, determining the critical bifurcation stretch and mode number by a linear incremental analysis when the two lateral surfaces are traction-free. Ogden and Chadwick [22] extended the controllable-deformation family to combined torsion–extension of solid cylinders and tubes, providing explicit stress fields for arbitrary stored-energy functions. Haughton and Ogden [23,24] established the general uniform axisymmetric primary state for inflated and axially loaded incompressible tubes, expressing the resultant axial force and pressure through derivatives of an arbitrary strain-energy function, and then used this state as the pre-bifurcation configuration for combined axial-force and pressure loading. Pan and Beatty [25] re-derived the uniform-compression end-load relation for Mooney–Rivlin rubber and confirmed that the incremental characteristic roots are always real and positive for Mooney–Rivlin materials under the empirical inequalities, and that the asymptotic axisymmetric buckling stretch for long tubes or solid rods approaches $\lambda_{z}$ ≈ 0.4437. Liu [26] considered the axisymmetric compression of a packer rubber tube sliding against a rigid casing, with the inner surface traction-free, and derived the primary axisymmetric deformation and stress fields for a neo-Hookean material. These classical and semi-classical solutions provide exact insight into the primary deformation state, but they do not represent the full packer setting process, in which the tube gradually contacts the casing, and then the mandrel, and in which interface friction produces non-uniform axial deformation and end bulging.

Recognizing the limitations of linear analytical approaches, a growing body of research has turned to FE simulation to capture the large deformation, contact nonlinearity, and material hyper-elasticity inherent in packer rubber compression. Many studies first characterize the hyper-elastic constitutive behavior of rubber and then employ numerical methods—typically FE analysis—to evaluate the sealing performance of packer rubber cylinders under axial compression and contact constraints [27,28].

Hu et al. [29] conducted a comprehensive FE study on the influence of rubber material on sealing performance, using the Mooney–Rivlin model calibrated from uniaxial tests. Zheng et al. [30] performed a coupled thermal–mechanical FE analysis to quantify the effect of stress relaxation on contact pressure degradation at 120 °C. Zuo et al. [31] analyzed the degradation of sealing performance caused by the stress relaxation effect using both theoretical analysis and FEM. Hu et al. [32] investigated the effects of stress relaxation and geometric parameters on packer sealing performance using an FE axisymmetric model. Jin et al. [33] studied the effects of temperature and the initial setting pressure on the sealing effects of packers based on FEM. Wang et al. [34] specifically examined the role of interface friction in a compression packer FE model, concluding that the friction coefficient significantly alters the axial stress redistribution and mid-height radial bulging. They also optimized the structure of the packer’s shoulder and the protective ring of the rubber barrels through FEM [35]. Wang et al. [36] investigated the failure mechanisms of packer rubber elements induced by casing deformation, including eccentricity, ovality, inner diameter variation, and inclination.

While these FE studies provide valuable quantitative insights, they are inherently case-specific: each simulation run is tied to a particular geometry, material parameter set, and loading path. No closed-form analytical framework exists that can predict the stress evolution under a large-deformation hyper-elastic constitutive law.

This paper aims to fill this gap by presenting a complete analytical solution for the axisymmetric compression of rubber cylinder based on the incompressible Mooney–Rivlin hyper-elastic model. The solution respects the exact finite-strain kinematics imposed by volume incompressibility and derives the full stress field ($\sigma_{r}$, $\sigma_{\theta }$, $\sigma_{z}$) through integration of the radial equilibrium equation with proper treatment of the hydrostatic pressure Lagrange multiplier. The frictionless analytical results are validated against FE simulations with excellent agreement.

## 2. The Complexity and Simplification of Rubber Cylinder Deformation

### 2.1. Structure of Rubber Cylinder

**Figure 1:**
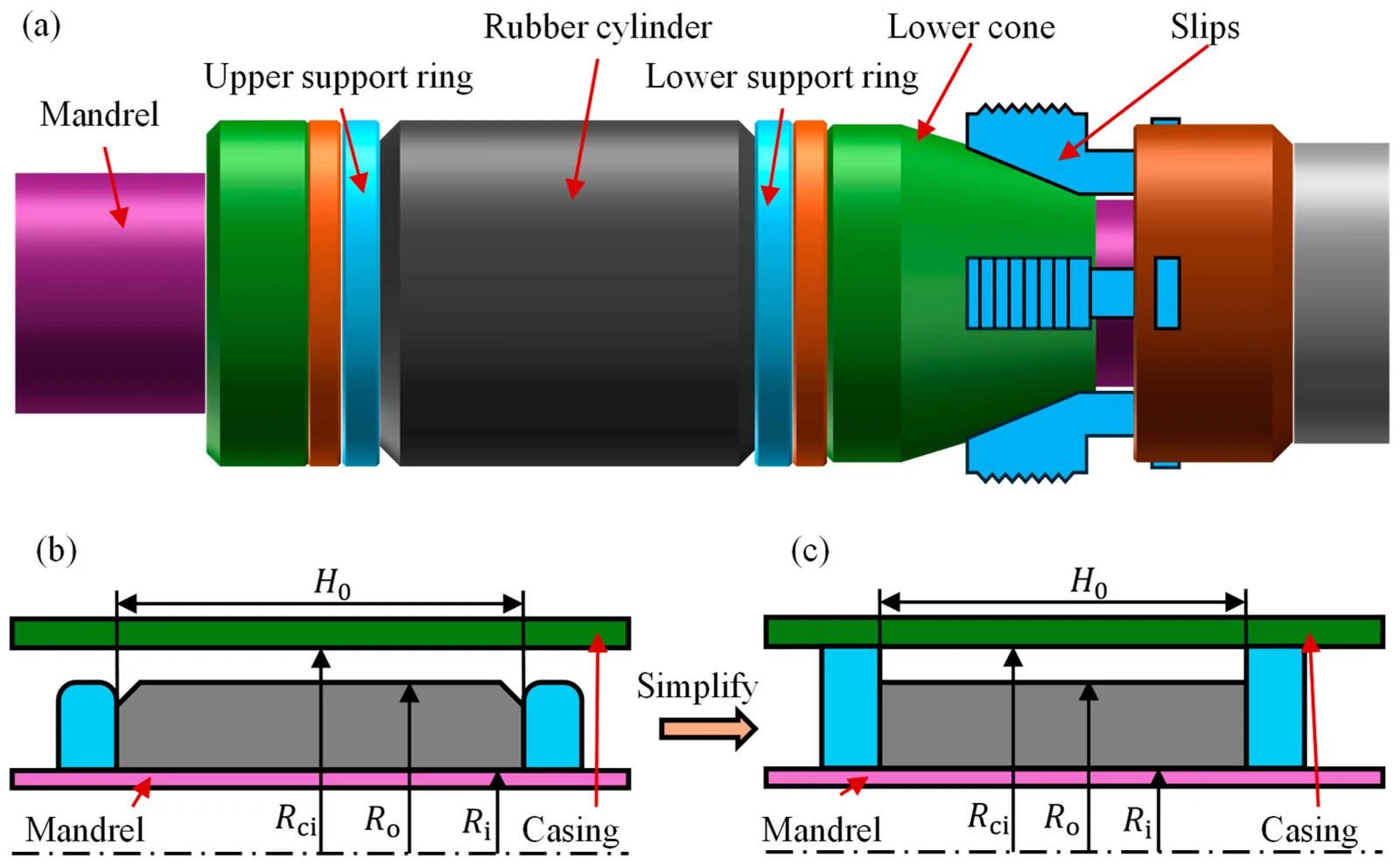
Composition and dimensions of the packer rubber cylinder. (**a**) Main components of the packer; (**b**) the structure and dimensions of the rubber cylinder before simplification; (**c**) the structure of the rubber cylinder after simplification.

The main structure of the packer is composed of upper and lower support ring, casing, mandrel, rubber cylinder, lower cone and slips [37], as shown in Figure 1a. Upper and lower support ring are used to compress the rubber cylinder. The center tube provides the necessary support and fluid passage to withstand complex downhole loads. The rubber cylinder of a packer serves as the core sealing element that achieves reliable annular isolation between the casing and the mandrel by radially expanding and maintaining sufficient contact stress against the casing wall under axial compression. The lower cone converts axial motion into radial movement of the slips, anchoring them against the casing to prevent axial displacement of the packer.

To obtain a theoretical solution, the geometric structures of the rubber cylinder and support rings are simplified in this paper. The inner wall of the support ring is assumed to align with the outer wall of the mandrel, and the outer wall aligns with the inner wall of the casing. The chamfers of the rubber cylinder are neglected, as shown in Figure 1b,c.

### 2.2. Deformation Process of the Rubber Cylinder

During packer operation, the lower support ring is fixed. An axial force acts on the upper support ring, making the rubber cylinder expand radially. As the axial force grows, the rubber cylinder expands gradually and finally touches the casing. When the axial force increases further, the contact force between the rubber and the casing becomes larger than the pressure difference across the seal, and then the packer is set [38].

**Figure 2:**
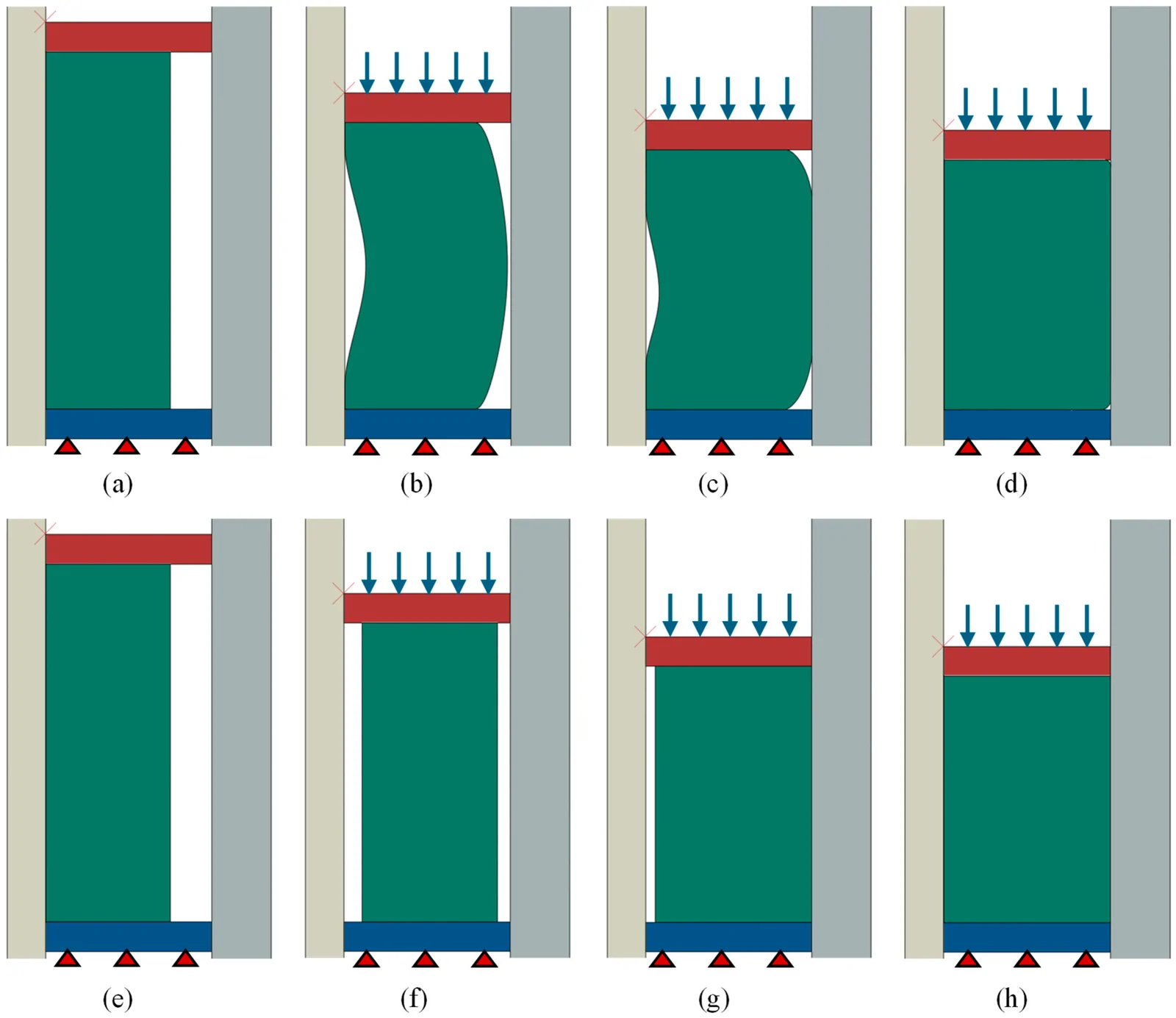
Deformation process of rubber cylinder: (**a**–**d**) with friction; (**e**–**h**)without friction. (**a**,**e**) Initial state; (**b**,**f**) free expansion stage; (**c**,**g**) constrained deformation upon contact with the casing; (**d**,**h**) fully constrained deformation stage.

Figure 2 presents the deformations of the simplified rubber cylinder with and without friction. It can be seen that the deformation process of the rubber cylinder can be divided into three stages:

(a) Free expansion stage. In this stage, the inner and outer walls of the rubber cylinder do not contact the casing or the mandrel. With friction, the ends of rubber cylinder undergo very small displacement due to the friction force from the support rings, while the middle part of the rubber cylinder experiences buckling deformation caused by axial compression. Without friction, both the inner and outer diameters increase simultaneously due to the Poisson effect.

(b) Constrained deformation stage under contact with the casing. As the inner and outer diameters increase during the free expansion stage, the rubber cylinder first contacts with the inner wall of the casing. Subsequently, it deforms under the constraint of the casing. Regardless of whether friction is present, the inner diameter decreases.

(c) Fully constrained deformation stage. As the inner diameter of the rubber cylinder continues to decrease, the inner wall of the rubber cylinder also comes into contact with the outer wall of the mandrel. The rubber cylinder then deforms within the enclosed space formed by the support rings, the inner wall of the casing, and the outer wall of the mandrel.

The deformation of the rubber cylinder with friction is much more complex than that without friction. To obtain a closed-form solution, this paper mainly studies the frictionless case.

## 3. Theoretical Formulation

### 3.1. Basic Assumptions

To simplify the theorical analysis, the following assumptions are adopted:

(1) The material is homogeneous, isotropic, and incompressible.

(2) The supporting rings, casing and mandrel are rigid and frictionless. Based on the frictionless assumption, there is no tangential constraint at the contact surfaces between the rubber cylinder and the supporting rings. The rubber can expand freely in the radial direction. Therefore, the deformation at the ends is identical to that in the middle, and the entire rubber cylinder deforms uniformly.

(3) Deformation is axisymmetric and uniform along the axial direction. Since the supporting rings are frictionless and sufficiently large, the deformation state is uniform throughout the rubber cylinder. That is, points originally on the same horizontal plane remain on the same plane after compression; points originally at the same radial position remain at the same radial position after deformation.

(4) Body forces are negligible.

The above assumptions warrant further discussion regarding their physical justification and influence on the model’s applicability to real-world packer systems.

(1) The assumption of homogeneous, isotropic, and incompressible material is widely accepted for HNBR rubber in the typical pressure range of compression packers (≤100 MPa). Although real compounds may exhibit slight anisotropy due to filler orientation and some compressibility under extreme confining pressures, the incompressible Mooney–Rivlin model is adequate for engineering design.

(2) The rigid-body assumption for the casing, mandrel, and support rings is physically justified because these steel components have an elastic modulus approximately three orders of magnitude higher than that of the rubber compound. Their deformation under the contact pressure is negligible compared to the large deformation of the rubber cylinder, and thus this assumption introduces no appreciable error.

(3) The assumption of axisymmetric and uniform axial deformation holds strictly only under frictionless conditions. When friction is present, the deformation becomes non-uniform along the axis (refer to Section 4), as will be shown in the numerical results. Nevertheless, the average contact pressure on the rubber–mandrel interface still agrees well with the theoretical prediction, justifying the use of the frictionless solution as a conservative engineering estimate.

(4) The frictionless assumption eliminates tangential tractions at contact surfaces. Under frictional conditions, the rubber cylinder exhibits end bulging and incomplete filling of the annular space, leading to deviations in the local contact pressure distribution. Despite these local effects, the overall relationship between the average contact pressure and the axial force remains approximately linear (refer to Section 4), supporting the practical utility of the theoretical result.

### 3.2. Geometry Relations

#### 3.2.1. Reference and Current Configurations

A cylindrical coordinate system $\left(R,\Theta ,Z\right)$ is used to describe the undeformed configuration, and (r,θ,z) for the deformed configuration, with the Z- and z-axes coinciding with the cylinder axis. Because the deformation is axisymmetric and free of shear, the principal directions remain radial, circumferential, and axial.

**Figure 3:**
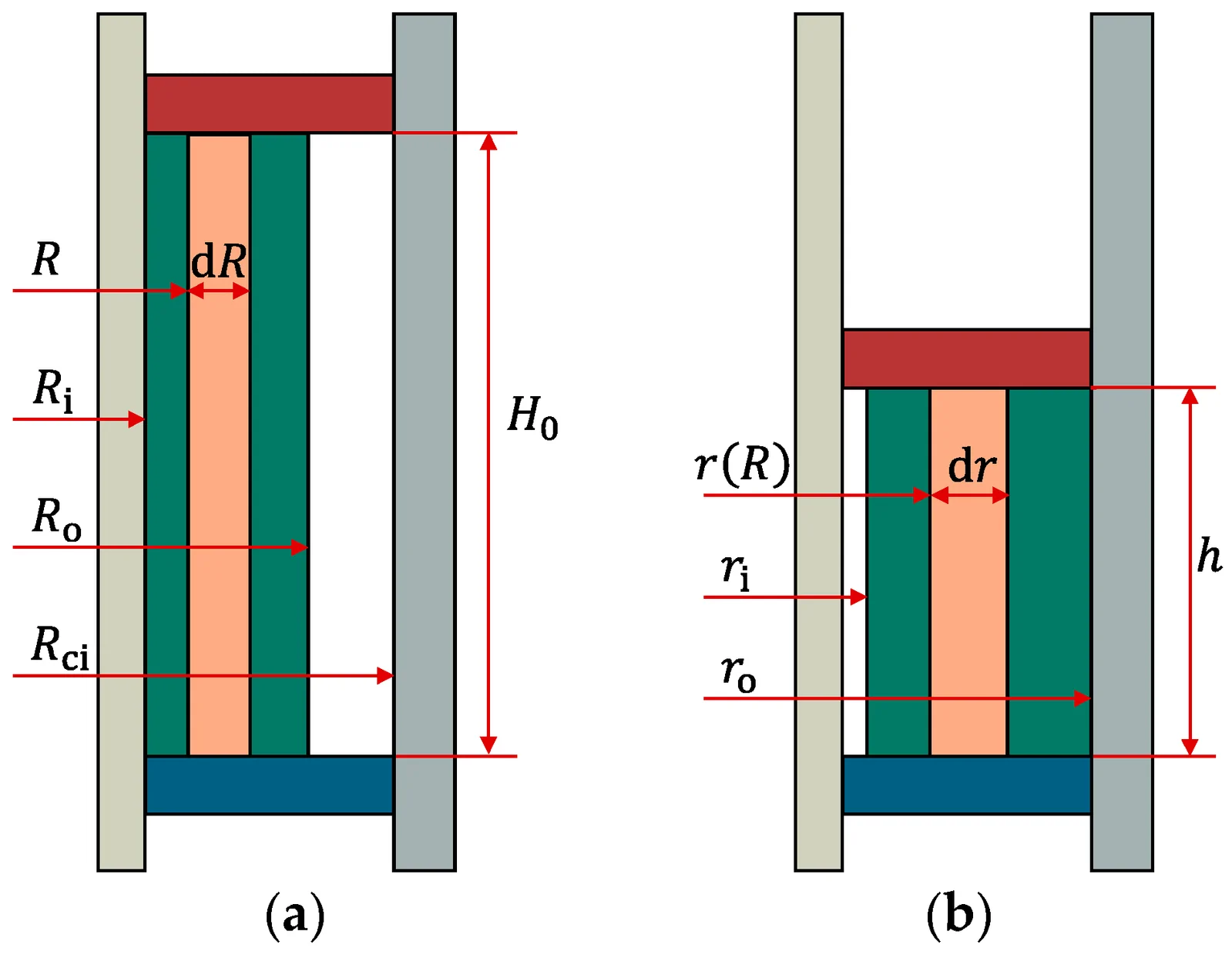
Main dimensions during the deformation process of the rubber cylinder. (**a**) Dimensions for initial reference configuration; (**b**) dimensions for current configuration.

The undeformed rubber cylinder is taken as the reference configuration, with its dimensions shown in Figure 3a, where $R_{i}$ is the inner radius of the undeformed rubber cylinder, $R_{o}$ is the outer radius of the undeformed rubber cylinder, R is the inner radius of an infinitesimal annular element arbitrarily selected within the undeformed rubber cylinder, and dR is the thickness of this infinitesimal annular element. The deformed rubber cylinder is shown in Figure 3b, referred to as the current configuration. In this figure, $r_{i}$ is the inner radius of the deformed rubber cylinder, $r_{o}$ is the outer radius of the deformed rubber cylinder, r is the inner radius of the deformed infinitesimal annular element corresponding to the dR element, and dr is the deformed thickness.

#### 3.2.2. Principal Stretches

Let a material point initially at radius R move to radius r(R) after deformation, while its axial and circumferential coordinates transform as $Z\to z=\lambda_{z}Z$ and Θ→θ=Θ (no circumferential slip). The three principal stretches are then defined in the Lagrangian description as

(1) $$\lambda_{r}\left(R\right)=\frac{dr}{dR},\;\;\;\lambda_{\theta }\left(R\right)=\frac{r\left(R\right)}{R},\;\;\;\lambda_{z}=\frac{h}{H_{0}}$$

where R is the radial material coordinate in the reference state. The axial stretch $\lambda_{z}$ is a quantity independent of R due to the uniform end-plate compression; the circumferential and radial stretches vary with R through the mapping r(R). For an incompressible rubber, the three stretches satisfy.

(2) $$J=\lambda_{r}\lambda_{\theta }\lambda_{z}\equiv 1$$

#### 3.2.3. Geometric Analysis for the Free Expansion Stage

For the free expansion stage, the deformation is unconstrained in the direction perpendicular to the axis. Since the material is homogeneous and isotropic, the radial and circumferential stretches are equal: $\lambda_{r}=\lambda_{\theta }$. Combined with the incompressibility condition $\lambda_{z}\lambda_{\theta }\lambda_{r}=1$, the following expression can be yielded

(3) $$\lambda_{r}=\lambda_{\theta }=\lambda_{z}^{-1/2}$$

The radial mapping follows directly as

(4) $$r\left(R\right)=R\lambda_{\theta }=\frac{R}{\sqrt{\lambda_{z}}}$$

When the deformed outer diameter of the rubber cylinder $r\left(R_{o}\right)$ equals the inner diameter of the casing $R_{ci}$, the free expansion stage ends. The axial stretch at this critical point can be calculated as

(5) $$r\left(R_{o}\right)=R_{ci}\to \lambda_{zcr1}=\frac{R_{o}^{2}}{R_{ci}^{2}}$$

#### 3.2.4. Geometric Analysis for the Deformation Stage Constrained by the Casing

During the deformation stage constrained by the casing, the stretches perpendicular to the axis, $\lambda_{r}$ and $\lambda_{\theta }$, are no longer equal. However, the incompressibility condition still holds. Substituting Equation (1) into Equation (2) yields

(6) $$\frac{dr}{dR}\frac{r\left(R\right)}{R}\lambda_{z}=1$$

Rearranging gives the differential equation

(7) $$r\left(R\right)dr=\frac{1}{\lambda_{z}}RdR$$

integrating from an arbitrary radius R (where $r=r\left(R\right)$) to the outer boundary $R=R_{o}$ (where $r=r_{o}$) yields

(8) $$\int_{r\left(R\right)}^{r_{o}}rdr=\frac{1}{\lambda_{z}}\int_{R}^{R_{o}}RdR$$

Evaluating the integrals, the radial mapping can be expressed explicitly as

(9) $${\left(r\left(R\right)\right)}^{2}=r_{o}^{2}-\frac{R_{o}^{2}-R^{2}}{\lambda_{z}}$$

Once the outer surface of rubber cylinder contacts the casing, the outer radius becomes fixed at $r_{o}=R_{ci}$. The inner radius $r_{i}$ continues to decrease with further compression. The radial mapping becomes

(10) $${\left(r\left(R\right)\right)}^{2}=R_{ci}^{2}-\frac{R_{o}^{2}-R^{2}}{\lambda_{z}}$$

#### 3.2.5. Geometric Relation for the Fully Constrained Stage

In the fully constrained deformation stage, it is assumed that the rubber cylinder is constrained in a regular annular space. If the mandrel and casing are treated as rigid bodies, the stretch increments in all three directions during this stage are one, that is,

(11) $$\lambda_{r}^{\prime }=\lambda_{\theta }^{\prime }=\lambda_{z}^{\prime }=1$$

### 3.3. Mooney–Rivlin Constitutive Model

Rubber cylinder is commonly described using the two-parameter Mooney–Rivlin model for hyper-elasticity. The strain energy density function is

(12) $$W=C_{10}\left(I_{1}-3\right)+C_{01}\left(I_{2}-3\right)$$

where $C_{10}$ and $C_{01}$ are material constants; and $I_{1}$, $I_{2}$ are the invariants of the right Cauchy–Green deformation tensor. These strain invariants can be expressed as

(13) $$I_{1}=\lambda_{r}^{2}+\lambda_{\theta }^{2}+\lambda_{z}^{2},\;\;I_{2}=\lambda_{r}^{2}\lambda_{\theta }^{2}+\lambda_{\theta }^{2}\lambda_{z}^{2}+\lambda_{z}^{2}\lambda_{r}^{2}$$

According to Equation (2), $I_{2}$ in Equation (13) can be expressed as

(14) $$I_{2}=\lambda_{r}^{-2}+\lambda_{\theta }^{-2}+\lambda_{z}^{-2}$$

For the incompressible material, the volume ratio J remains constant at 1. By enforcing this incompressible constraint to the strain energy function, we can obtain

(15) $$\bar{W}=C_{10}\left(I_{1}-3\right)+C_{01}\left(I_{2}-3\right)-p\left(J-1\right)$$

where p is an undetermined Lagrange multiplier, which needs to be determined from the equilibrium equations and boundary conditions.

The principal Cauchy stress $\sigma_{i}$ in the elongated direction can be expressed as

(16) $$\sigma_{i}=J^{-1}\lambda_{i}\frac{\partial \bar{W}}{\partial \lambda_{i}}=-p+2\left(C_{10}\lambda_{i}^{2}-C_{01}\lambda_{i}^{-2}\right),\;\;\left(i=r,\theta ,z\right)$$

### 3.4. Stresses for Different Stages

#### 3.4.1. Stress Analysis for the Free Expansion Stage

During the free expansion stage, the radial and circumferential deformations are unconstrained, so the radial and circumferential stresses are zero: $\sigma_{r}=\sigma_{\theta }=0$. According to Equations (3) and (16), the hydrostatic pressure is obtained as

(17) $$p=2(C_{10}\lambda_{z}^{-1}-C_{01}\lambda_{z})$$

The axial stress then follows as

(18) $$\sigma_{z}=2\left(C_{10}+\frac{C_{01}}{\lambda_{z}}\right)\left(\lambda_{z}^{2}-\lambda_{z}^{-1}\right)$$

When $\lambda_{z}\leq \lambda_{zcr}$, the axial force is given by

(19) $$F_{z1}=\sigma_{z}A=2\pi \left(R_{o}^{2}-R_{i}^{2}\right)\left(\lambda_{z}-\lambda_{z}^{-2}\right)\left(C_{10}+\frac{C_{01}}{\lambda_{z}}\right)$$

where A is the cross-sectional area of the deformed rubber cylinder.

#### 3.4.2. Stress Analysis for the Deformation Stage Constrained by the Casing

Under axisymmetric, body-force-free, and frictionless conditions, the radial equilibrium equation is

(20) $$\frac{d\sigma_{r}}{dr}+\frac{\sigma_{r}-\sigma_{\theta }}{r}=0$$

Substituting the expressions for $\lambda_{r}$ and $\lambda_{\theta }$ from Equation (1) into $\sigma_{r}$ and $\sigma_{\theta }$ in Equation (16), and then substituting the result into Equation (20), yields

(21) $$\frac{d\sigma_{r}}{dr}=\frac{2C_{10}}{r}\left(\frac{r^{2}}{R^{2}}-\frac{R^{2}}{\lambda_{z}^{2}r^{2}}\right)-\frac{2C_{01}}{r}\left(\frac{R^{2}}{r^{2}}-\lambda_{z}^{2}\frac{r^{2}}{R^{2}}\right)$$

Using $\lambda_{r}=dr/dR$ from Equations (1), (6) and (21) becomes

(22) $$d\sigma_{r}=\frac{2C_{10}}{\lambda_{z}}\left(\frac{1}{R}-\frac{1}{\lambda_{z}^{2}}\frac{R^{3}}{r^{4}}\right)dR-\frac{2C_{01}}{\lambda_{z}}\left(\frac{R^{3}}{r^{4}}-\frac{\lambda_{z}^{2}}{R}\right)dR$$

Substituting Equation (10) into Equation (22) and integrating gives

(23) $$\sigma_{r}=\frac{C_{10}+C_{01}\lambda_{z}^{2}}{\lambda_{z}}\left(\ln\frac{R^{2}}{\lambda_{z}r^{2}}-\frac{R_{ci}^{2}\lambda_{z}-R_{o}^{2}}{\lambda_{z}r^{2}}\right)+C_{5}$$

where $C_{5}$ is integral constant determined by boundary condition. The boundary condition for this stage is $\sigma_{r}(R_{i})=0$ (where $r_{i}$ corresponds to $R_{i}$). Substituting this into the above expression yields

(24) $$C_{5}=\frac{C_{10}+C_{01}\lambda_{z}^{2}}{\lambda_{z}}\left(\ln\frac{\lambda_{z}r_{i}^{2}}{R_{i}^{2}}+\frac{R_{ci}^{2}\lambda_{z}-R_{o}^{2}}{\lambda_{z}r_{i}^{2}}\right)$$

From Equation (16) the Lagrange multiplier p is obtained as

(25) $$p=-\sigma_{r}+2\left(C_{10}\frac{R^{2}}{\lambda_{z}^{2}r^{2}}-C_{01}\lambda_{z}^{2}\frac{r^{2}}{R^{2}}\right)$$

Finally, the axial stress $\sigma_{z}$ and circumferential stress $\sigma_{\theta }$ can be determined as

(26) $$\left\{\begin{array}{l} \sigma_{z}=-p+2\left(C_{10}\lambda_{z}^{2}-C_{01}\lambda_{z}^{-2}\right)\\ \sigma_{\theta }=-p+2\left(C_{10}\frac{r^{2}}{R^{2}}-C_{01}\frac{R^{2}}{r^{2}}\right) \end{array}\right.$$

When $\lambda_{zcr1}<\lambda_{z}<\lambda_{zcr2}$, integrating $\sigma_{z}$ in Equation (26) gives the axial force

(27) $$\begin{array}{cc} F_{z2}=\int_{r_{i}}^{r_{o}}\sigma_{z}\left(r\right)dA & =\frac{1}{\lambda_{z}}\int_{R_{i}}^{R_{o}}\sigma_{z}\left(R\right)dA_{0}\\ & =\pi \left(\mu_{\lambda }R_{o}^{2}+2C_{01}A_{\lambda }\right)\left(\ln\frac{R_{o}^{2}}{R_{i}^{2}}+\ln\frac{r_{i}^{2}}{R_{ci}^{2}}\right)+\pi \frac{\mu_{\lambda }A_{\lambda }}{\lambda_{z}r_{i}^{2}}\left(R_{o}^{2}-R_{i}^{2}\right)+F_{z1} \end{array}$$

where $r_{i}^{2}=R_{ci}^{2}-\frac{R_{o}^{2}-R_{i}^{2}}{\lambda_{z}}$. Additionally, $\mu_{\lambda }=\frac{C_{10}+C_{01}\lambda_{z}^{2}}{\lambda_{z}^{2}}$ is introduced as an effective shear modulus that depends on the current axial stretch $\lambda_{z}$. It reduces to the classical shear modulus $C_{10}+C_{01}$ in the undeformed state and governs the logarithmic variation in the radial stress in the casing-constrained stage. We also introduce the geometric quantity

(28) $$A_{\lambda }=R_{ci}^{2}\lambda_{z}-R_{o}^{2}$$

When $\lambda_{z}=\lambda_{zcr1}$, $A_{\lambda }=0$ and $\ln\frac{R_{o}^{2}}{R_{i}^{2}}+\ln\frac{r_{i}^{2}}{R_{ci}^{2}}=0$. Therefore, $F_{z2}\left(\lambda_{zcr1}\right)=F_{z1}\left(\lambda_{zcr1}\right)$, indicating that the axial forces in the two stages are continuous. The axial stretch at the end of the deformation stage constrained by the casing is

(29) $$\lambda_{zcr2}=\frac{R_{o}^{2}-R_{i}^{2}}{R_{ci}^{2}-R_{i}^{2}}$$

#### 3.4.3. Stress Analysis for the Fully Constrained Stage

During the fully constrained stage, according to Equations (11) and (16), the stress at each point inside the rubber cylinder can be obtained as

(30) $$\left\{\begin{array}{c} \sigma_{r}=-p+2\left(C_{10}\lambda_{rcr2}^{2}-C_{01}\lambda_{rcr2}^{-2}\right)\\ \sigma_{\theta }=-p+2\left(C_{10}\lambda_{\theta cr2}^{2}-C_{01}\lambda_{\theta cr2}^{-2}\right)\\ \sigma_{z}=-p+2\left(C_{10}\lambda_{zcr2}^{2}-C_{01}\lambda_{zcr2}^{-2}\right) \end{array}\right.$$

where $\lambda_{zcr2}$, $\lambda_{rcr2}$, and $\lambda_{\theta cr2}$ are the stretch ratios at the end of the previous stage. Furthermore, $\lambda_{rcr2}$ and $\lambda_{\theta cr2}$ are functions of both $\lambda_{zcr2}$ and the radius R,

(31) $$\left\{\begin{array}{c} \lambda_{rcr2}=\frac{R}{\sqrt{\lambda_{zcr2}^{2}R_{ci}^{2}-\lambda_{zcr2}\left(R_{o}^{2}-R^{2}\right)}}\\ \lambda_{\theta cr2}=\sqrt{\frac{R_{ci}^{2}}{R^{2}}-\frac{1}{\lambda_{zcr2}}\left(\frac{R_{o}^{2}}{R^{2}}-1\right)} \end{array}\right.$$

Due to incompressibility, as the external axial force continues to increase, the second term in $\sigma_{z}$ no longer changes; only p varies. From the previous calculations, it is known that p varies very little along the radial direction. Therefore, in the fully constrained stage, p can be determined from the applied external force $F_{z}$ and $p_{cr2}$, $F_{zcr2}$ at the end of the previous stage,

(32) $$p=p_{cr2}-\frac{F_{z}-F_{zcr2}}{\pi \left(R_{ci}^{2}-R_{i}^{2}\right)}$$

where $p_{cr2}$ is calculated from Equation (25) when $\lambda_{z}=\lambda_{zcr2}$. Since $p_{cr2}$ is a function of R, the resulting $\sigma_{z}$ still varies along the radial direction. The sign convention for $F_{z}$ and $F_{zcr2}$ in the above equation is that compression is negative.

In the fully constrained stage, $\left|\sigma_{r}\left(R_{i}\right)\right|$ represents the contact pressure between the rubber cylinder and the mandrel, and $\left|\sigma_{r}\left(R_{o}\right)\right|$ represents the contact pressure between the rubber cylinder and the casing. Since $\left|\sigma_{r}\left(R_{o}\right)\right|>\left|\sigma_{r}\left(R_{i}\right)\right|$, when $\left|\sigma_{r}\left(R_{i}\right)\right|$ exceeds the required sealing pressure difference Δp, the corresponding axial force $F_{z}$ is the axial thrust needed for designing the packer setting mechanism. The minimum axial force required to bear the pressure difference Δp is

(33) $$F_{z}=F_{zcr2}+\pi \left(R_{ci}^{2}-R_{i}^{2}\right)\left(p_{cr2}\left(R_{i}\right)+2\left(C_{10}{\left(\lambda_{rcr2}\left(R_{i}\right)\right)}^{2}-C_{01}{\left(\lambda_{rcr2}\left(R_{i}\right)\right)}^{-2}\right)-\Delta p\right)$$

According to Equation (32), the relationship between contact pressure and axial force can also be obtained as

(34) $$P_{c}=-\sigma_{r}\left(R_{i}\right)=p_{cr2}-\frac{F_{z}-F_{zcr2}}{\pi \left(R_{ci}^{2}-R_{i}^{2}\right)}-2\left(C_{10}{\left(\lambda_{rcr2}\left(R_{i}\right)\right)}^{2}-C_{01}{\left(\lambda_{rcr2}\left(R_{i}\right)\right)}^{-2}\right)$$

The above equation indicates that there is a proportional relationship between the contact pressure and the axial force.

Equation (34) has direct practical value for packer design. Since it requires only the geometric dimensions $\left(R_{i},R_{o},R_{ci},H_{0}\right)$ and the material parameter $\left({C_{01},C}_{10}\right)$, engineers can obtain a quick estimate of the required axial force for a target sealing pressure without performing numerical simulations.

Because the frictionless assumption eliminates tangential tractions at the contact surfaces, the analytical prediction serves as a conservative lower-bound estimate of the contact pressure under actual frictional service conditions. This conservatism provides a built-in safety margin for sealing performance assessment.

For practical design, it is recommended to apply an additional safety factor to account for uncertainties in material properties, friction conditions, and geometric tolerances. The specific value of the safety factor should be determined based on the operating environment and the required reliability level of the sealing system.

### 3.5. Remark on Stability

The present study focuses on the axisymmetric compression of packer rubber cylinders within the stable deformation regime, where the analytical solution remains valid. It is therefore necessary to clarify that the geometric and loading conditions considered herein lie outside the buckling range predicted by existing theories.

Wilkes [21] analyzed the bifurcation of a compressed hyper-elastic tube and established the critical compression ratio for the onset of axisymmetric instability. For thin-walled tubes, an approximate analytical expression is available; for thick-walled tubes and solid cylinders, the critical values are given graphically. Subsequently, Pan and Beatty [25] derived a closed-form condition for Mooney–Rivlin materials and showed that the long-tube limit of the axisymmetric critical stretch ratio is approximately 0.4437.

The maximum achievable axial stretch ratio under normal operating conditions can be expressed in dimensionless form by rewriting Equation (29) as:

(35) $$\lambda_{zcr2}=\frac{\rho_{0}^{2}-1}{\beta^{2}\rho_{0}^{2}-1}$$

where $\beta =R_{ci}/R_{o}$ is the expansion ratio and $\rho_{o}=R_{o}/R_{i}$ is the initial outer-to-inner radius ratio. For typical compression packers, β is generally less than 1.2 and $\rho_{0}$ ranges between 1.5 and 2.0. 

Based on the boundary values of β and $\rho_{0}$, the minimum value of $\lambda_{zcr2}$ is 0.7353. This value is considerably larger than the limit of the axisymmetric critical stretch ratio for Mooney–Rivlin materials ($\lambda_{zcr}$ = 0.4437, Pan and Beatty [25]). Therefore, the axisymmetric deformation mode assumed in the analytical derivation is stable for the present problem. A comprehensive stability analysis, which would require solving the full bifurcation problem for the specific geometry and material parameters, is beyond the scope of this study and is identified as a topic for future investigation.

## 4. Comparison with Finite Element Results

### 4.1. Finite Element Model

#### 4.1.1. Setup of the FE Model

**Figure 4:**
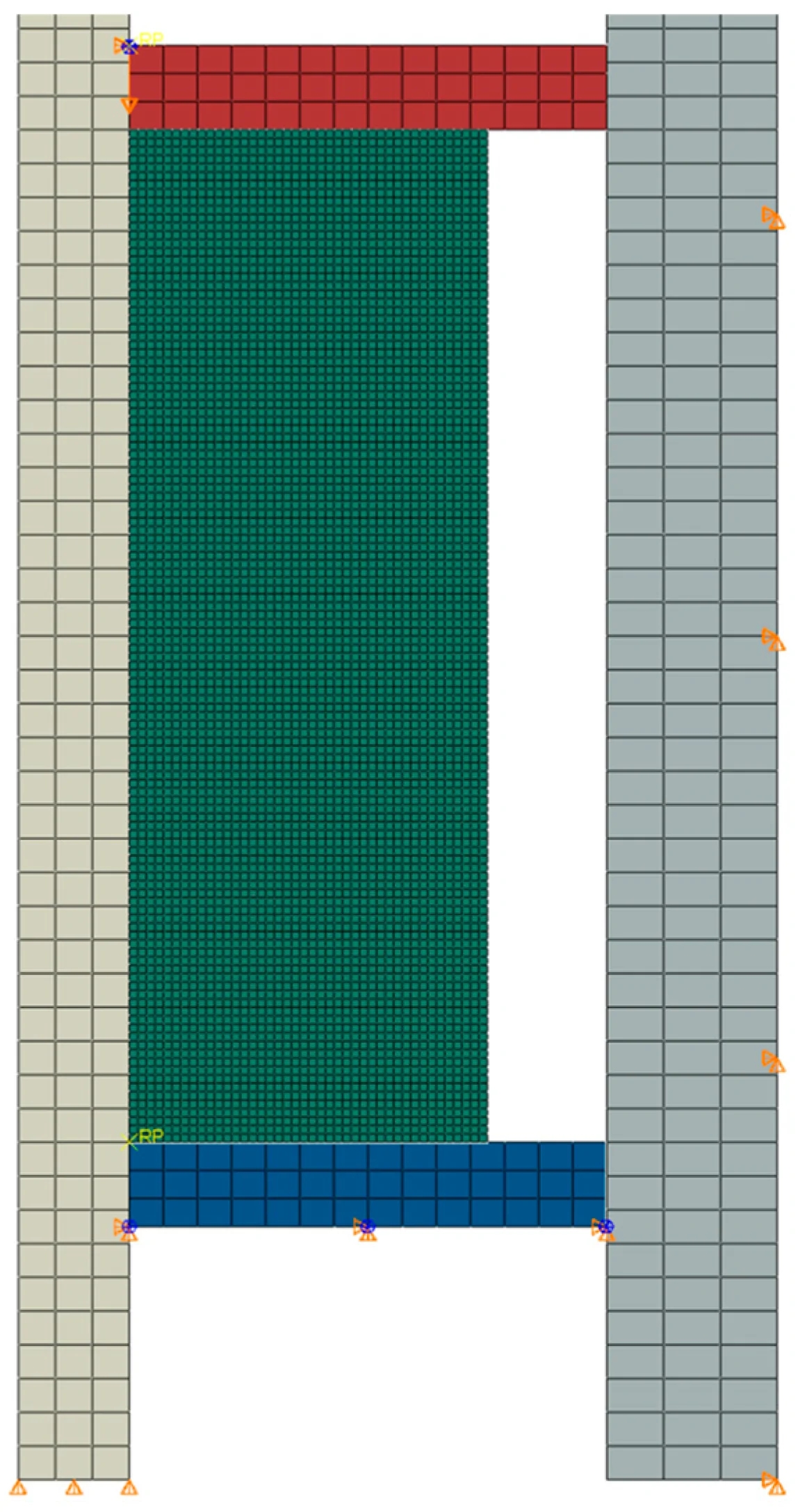
Finite element model for used for comparative study.

A two-dimensional axisymmetric FE model is developed to simulate the compression process of the rubber cylinder, as shown in Figure 4. The FE analysis was performed using a commercial FE software package with an implicit solver. The geometric dimensions and material parameters are consistent with those used in the theoretical analysis. The rubber cylinder is meshed with CAX4H elements (4-node bilinear axisymmetric quadrilateral, hybrid formulation) to accommodate the incompressible hyper-elastic behavior. The model consisted of approximately 5040~11,600 elements, with a refined global mesh size of 0.5 mm in the contact regions to accurately capture the stress gradients. Nonlinear geometry (NLGEOM) was enabled to account for large deformations.

The hyper-elastic material is modeled using the two-parameter Mooney–Rivlin model. The material constants $C_{10}$ = 2.3107 MPa and $C_{01}$ = 0.5777 MPa were adopted from the characterization of Shore-A 90 HNBR reported by Wei et al. [39], in which the same rubber hardness grade was employed.

The support rings, casing, and mandrel are modeled as rigid bodies by assigning a rigid body constraint to their respective regions, with the motion governed by a reference point at each component. Contact pairs are defined between the rubber cylinder and the surrounding components. For the frictionless cases, the friction coefficient is set to zero. A displacement-controlled axial compression is applied to the upper support ring, while the lower support ring is fixed. The axial displacement was applied incrementally, and the Newton–Raphson iterative method was employed to solve the nonlinear equilibrium equations at each increment, using default convergence tolerances.

The geometric parameters for the comparison are shown in Table 1.

#### 4.1.2. Mesh Convergence Study

To ensure that the finite element results are independent of mesh refinement, a mesh convergence study was conducted prior to the main simulations. Five mesh densities (2.0 mm, 1.0 mm, 0.5 mm, 0.25 mm, 0.125 mm) were tested under identical loading conditions: a prescribed axial displacement corresponding to a reaction force of 90 kN, with a friction coefficient of 0.3. The axial displacement of the upper support ring was monitored as the convergence criterion.

**Figure 5:**
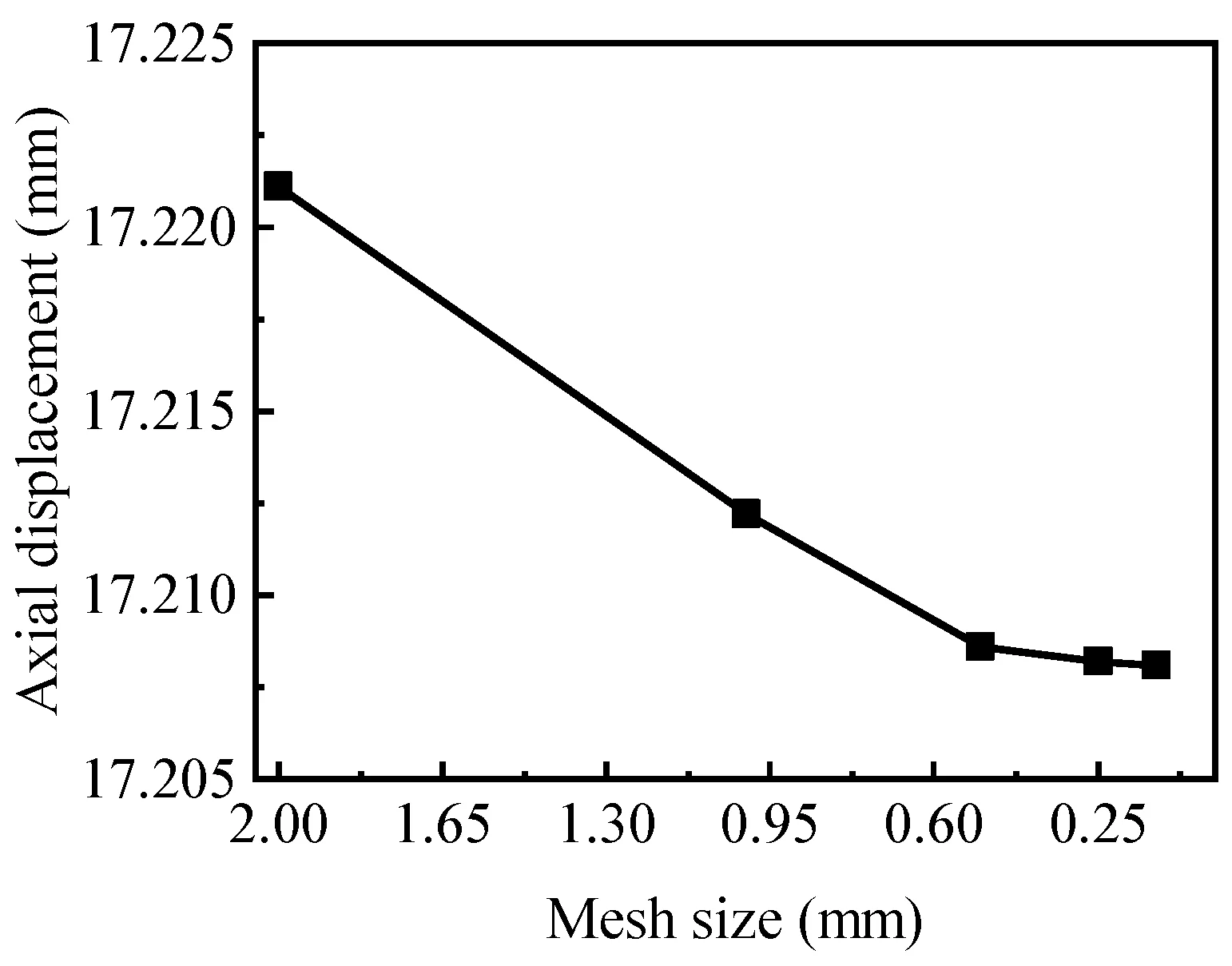
Mesh convergence analysis: axial displacement as a function of mesh size (axial force = 90 kN, friction coefficient = 0.3).

The results are shown in Figure 5. As shown in the figure, the axial displacement changes from about 17.2211 mm at a mesh size of 2.00 mm to about 17.2122 mm at 1.00 mm, 17.2086 mm at 0.50 mm, and then stabilizes around 17.208 mm for mesh sizes of 0.25 mm and 0.125 mm. The difference between the 0.50 mm mesh and the finest 0.125 mm mesh is less than 0.005 mm (below 0.03%), confirming that mesh convergence is achieved at a global element size of 0.50 mm.

Based on this study, a global element size of 0.5 mm was adopted for all subsequent simulations. Depending on the specific geometry, the total number of elements ranged from approximately 5040 to 11,600.

### 4.2. Comparison of Theorical and Numerical Results Under Frictionless Condition

#### 4.2.1. Comparison for the Free Expansion Stage

**Figure 6:**
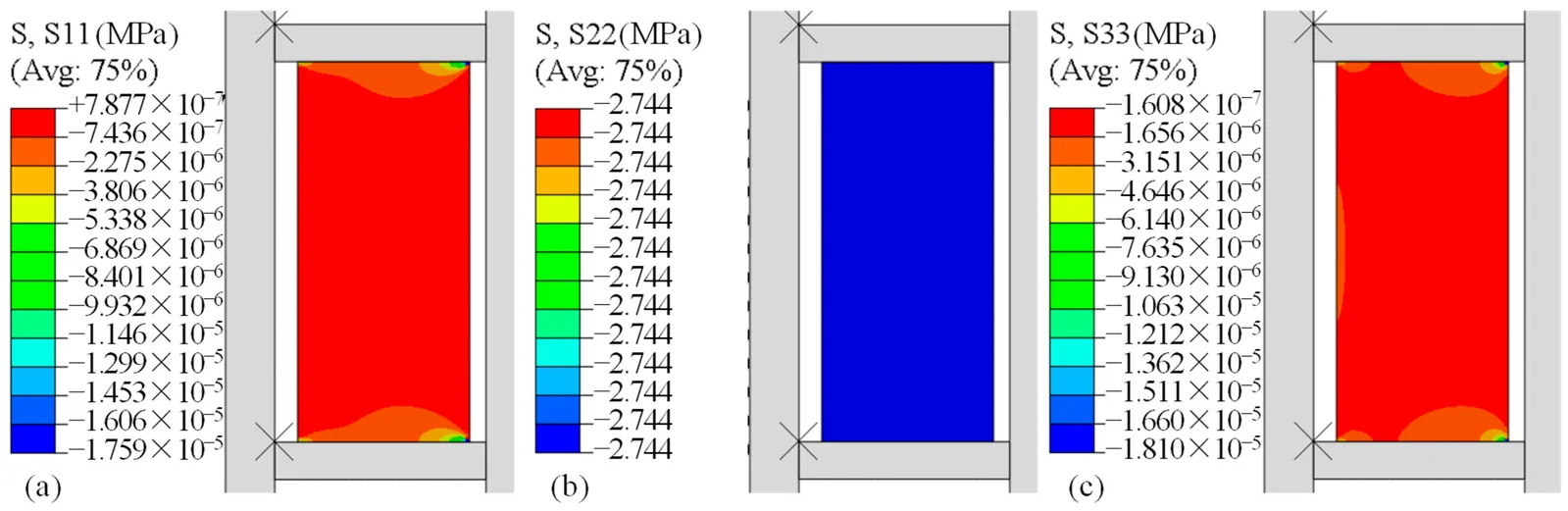
Stress distribution at a stretch ratio of 0.848 in the free expansion stage. (**a**) Radial stress; (**b**) axial stress; (**c**) circumferential stress.

Under frictionless conditions in the free expansion stage, the radial and circumferential stresses are theoretically zero throughout the entire domain, while the axial stress is nonzero and uniformly distributed. Figure 6 presents the finite element results at a stretch ratio of 0.848. S11 and S33 represent the radial and circumferential stresses, respectively. Except for stress concentrations at the four corners, the stresses are essentially uniformly distributed with magnitudes below $10^{-5}$ MPa, showing good agreement with the theoretical prediction. S22 represents the axial stress with a value of 2.744 MPa, which matches the theoretical result 2.753 MPa.

#### 4.2.2. Comparison for the Deformation Stage Constrained by the Casing

**Figure 7:**
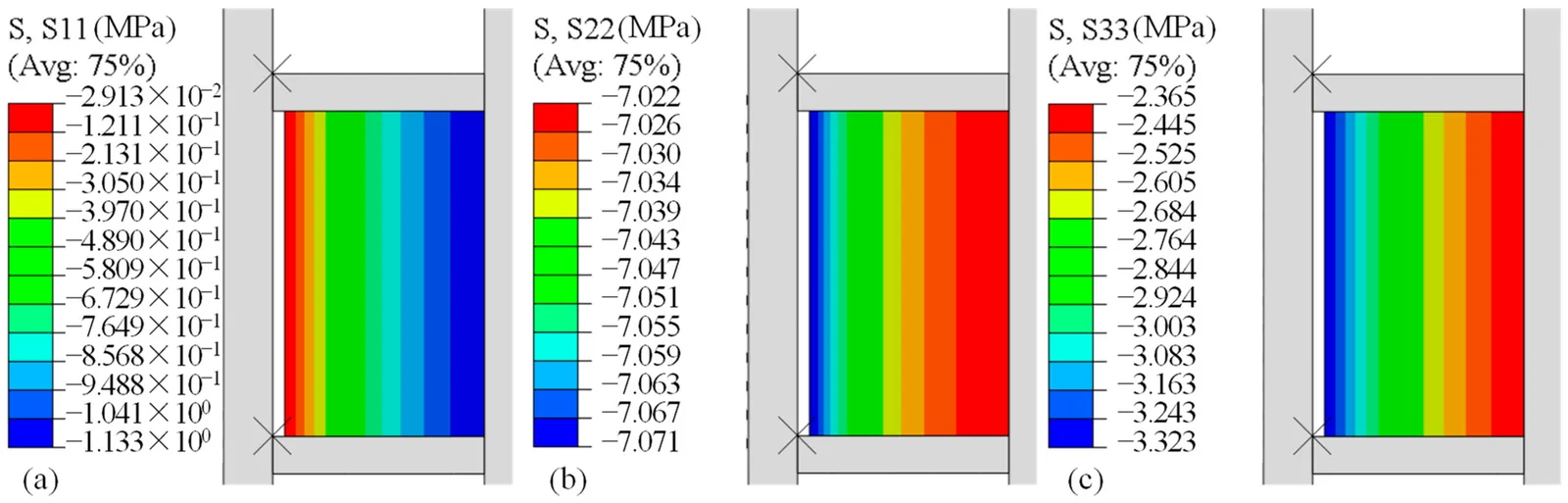
Stress distribution at a stretch ratio of 0.727 in the deformation stage constrained by the casing. (**a**) Radial stress; (**b**) axial stress; (**c**) circumferential stress.

Figure 7 shows the stress distributions in the three principal directions at a stretch ratio of 0.727 in the casing-constrained deformation stage under frictionless conditions. It can be seen that the stresses in this stage do not vary along the axial direction; that is, under frictionless conditions, the stresses are not functions of the z-coordinate. The stresses vary along the radial direction. The radial and circumferential stresses change significantly with radius, while the axial stress varies very little with radius.

**Figure 8:**
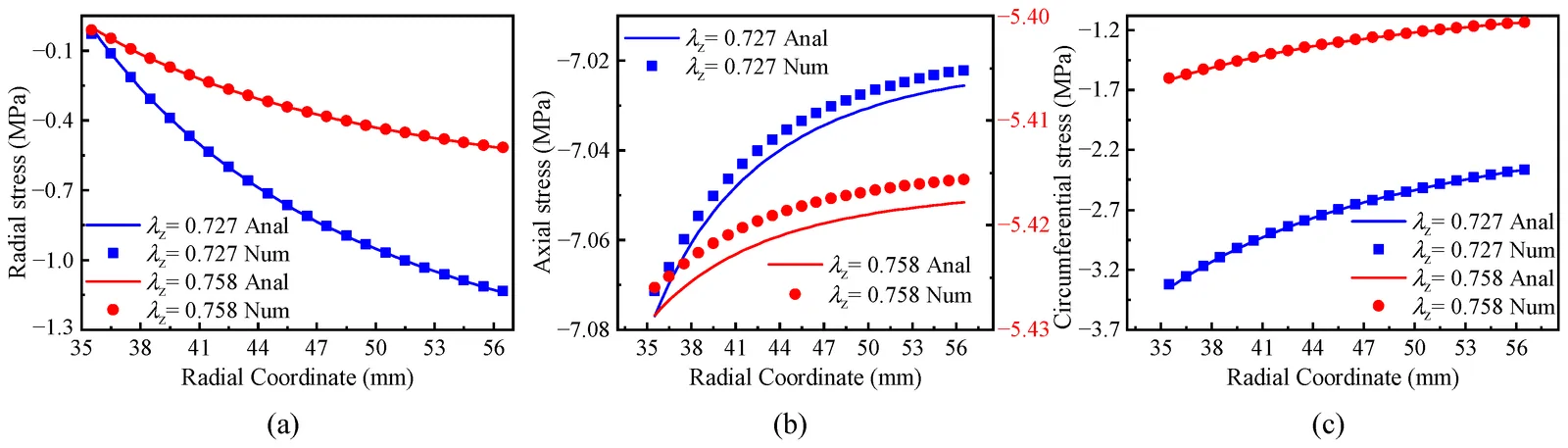
Comparison of theoretical and numerical stress results at different stretches in the deformation stage constrained by the casing. (**a**) Radial stress; (**b**) axial stress; (**c**) circumferential stress.

Figure 8 presents the comparison between theoretical and numerical stress results at different stretch ratios in the casing-constrained deformation stage under frictionless conditions. It can be observed that, under frictionless conditions, the theoretical and numerical results for the radial and circumferential stresses almost coincide at different stretch ratios. Although the theoretical and numerical results for the axial stress appear to differ considerably, the maximum relative error is actually 0.08%. Therefore, the theoretical results are in excellent agreement with the numerical results. This confirms the correctness of the theoretical formulas derived in this paper and also reflects the reliability of the finite element software.

**Figure 9:**
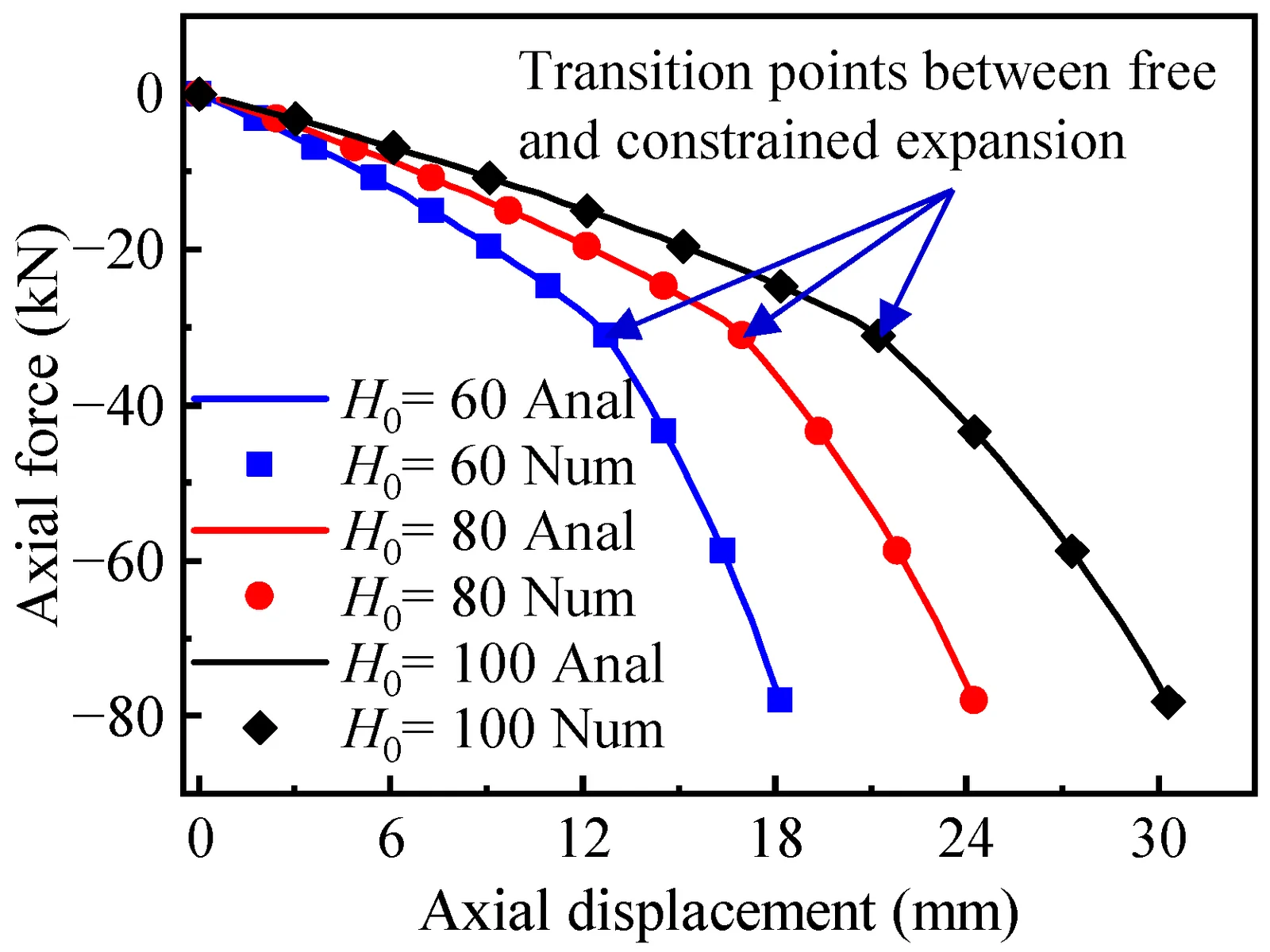
Comparison of theoretical and numerical results of axial force versus axial displacement for different rubber cylinder lengths under frictionless compression.

Figure 9 presents the comparison between theoretical and numerical results of axial force versus axial displacement for different rubber cylinder lengths under frictionless compression. It can be seen that the theoretical and numerical results of the axial force almost coincide, which verifies the correctness of the theoretical integration process. It can also be observed that the two stages of the rubber cylinder compression process have a clear transition point.

#### 4.2.3. Comparison for the Fully Constrained Stage

**Figure 10:**
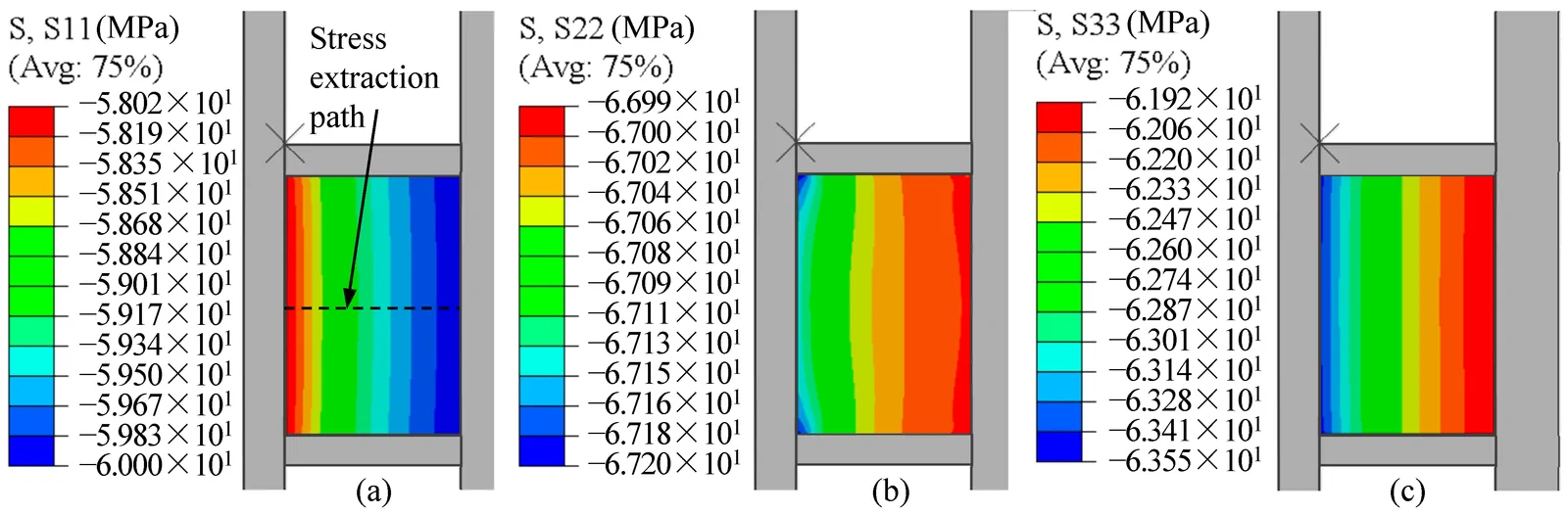
Stress distribution in the three principal directions at an axial force of Fz = 584.8 kN. (**a**) Radial stress; (**b**) axial stress; (**c**) circumferential stress.

In the fully constrained stage, the incompressible rubber cylinder undergoes no further deformation. Therefore, the calculation in this stage involves determining the stresses in all directions of the rubber cylinder for a given axial force. Figure 10 shows the stress distribution in the three principal directions of the rubber cylinder at an axial force of 584.8 kN. It can be seen from the figure that the stresses in all three directions vary along the radial direction. Due to the deformation of the casing and mandrel, the stresses in the three directions also vary slightly along the axial direction.

**Figure 11:**
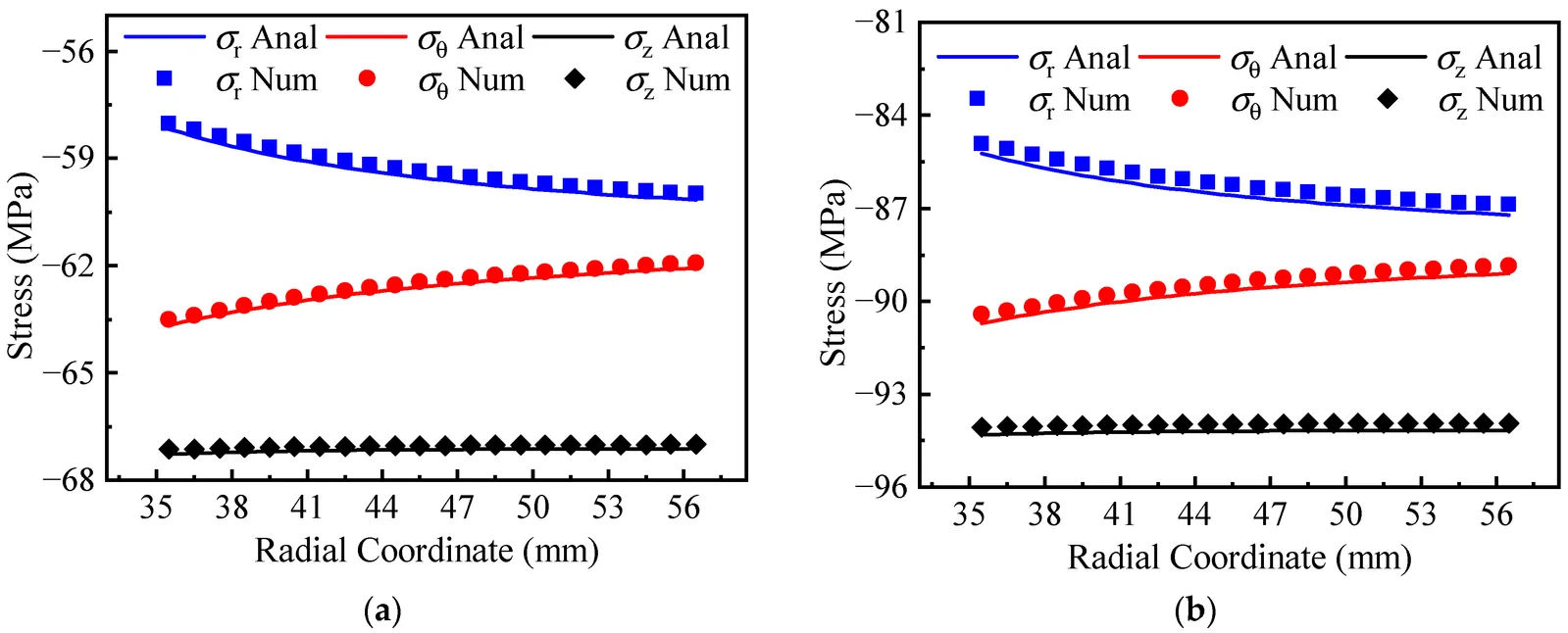
Radial distribution of stresses in the rubber cylinder under different axial forces. (**a**) $F_{z}$ = 584.8 kN; (**b**) $F_{z}$ = 820.3 kN.

Figure 11 presents the comparison between theoretical and numerical results of the stresses in each direction under different axial forces. Figure 11a is the comparison for $F_{z}=$ 584.8 kN. It can be seen that the theoretical and numerical results exhibit the same trends, with the axial stress varying the least along the radial direction. The theoretical results are slightly lower than the numerical results. The maximum relative error is approximately 0.36% for the radial stress, 0.31% for the circumferential stress, and 0.28% for the axial stress. Figure 11b shows the comparison for $F_{z}=$ 820.3 kN. It can be observed that the change in axial force does not alter the trend of the stress distribution. However, analyzing the difference between the theoretical and numerical stresses reveals that as the axial force increases, the relative error increases (the radial stress error increases from 0.36% to 0.41%).

### 4.3. Applicability of Theoretical Results to Frictional Conditions

**Figure 12:**
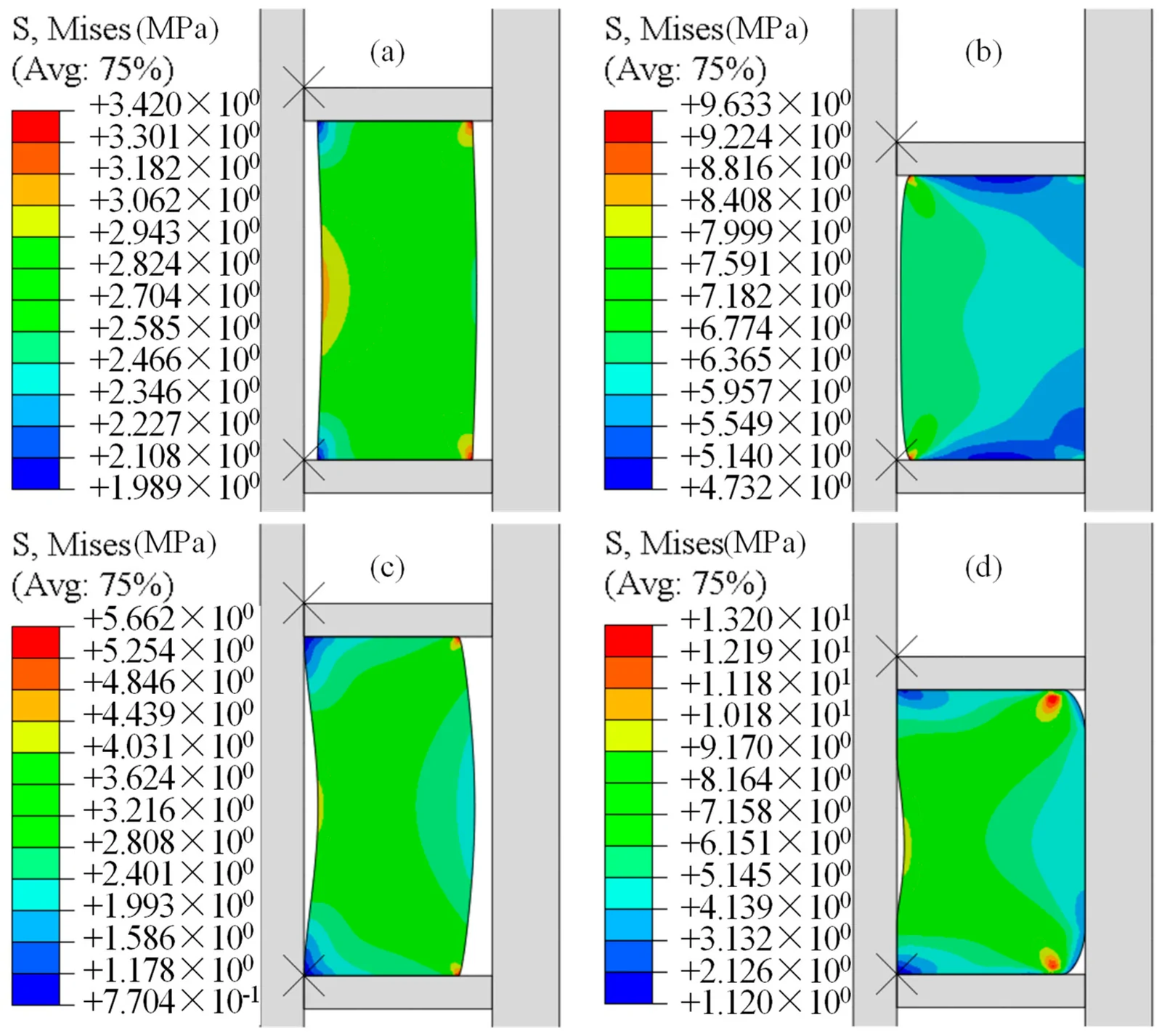
Deformations of the rubber cylinder under different stretch and friction coefficients. (**a**) $\lambda_{z}=0.5\lambda_{zcr2},f=0.05$; (**b**) $\lambda_{z}=0.95\lambda_{zcr2},f=0.05$; (**c**) $\lambda_{z}=0.5\lambda_{zcr2},f=0.15$; (**d**) $\lambda_{z}=0.95\lambda_{zcr2},f=0.15$.

Figure 12 shows the deformations of the rubber cylinder under different stretch ratios and friction coefficients. It can be seen that when friction is present, the deformation of the rubber cylinder is highly complex. Moreover, the deformation pattern varies with the friction coefficient. Due to the effect of friction, the rubber cylinder no longer maintains a perfect cylindrical shape during the free expansion stage but instead bulges outward. The larger the friction coefficient, the more severe the bulging. When the friction coefficient is 0.15, the rubber cylinder first contacts with the mandrel at the ends due to the frictional action of the support rings, and only after sufficient bulging does it begin to contact with the casing.

**Figure 13:**
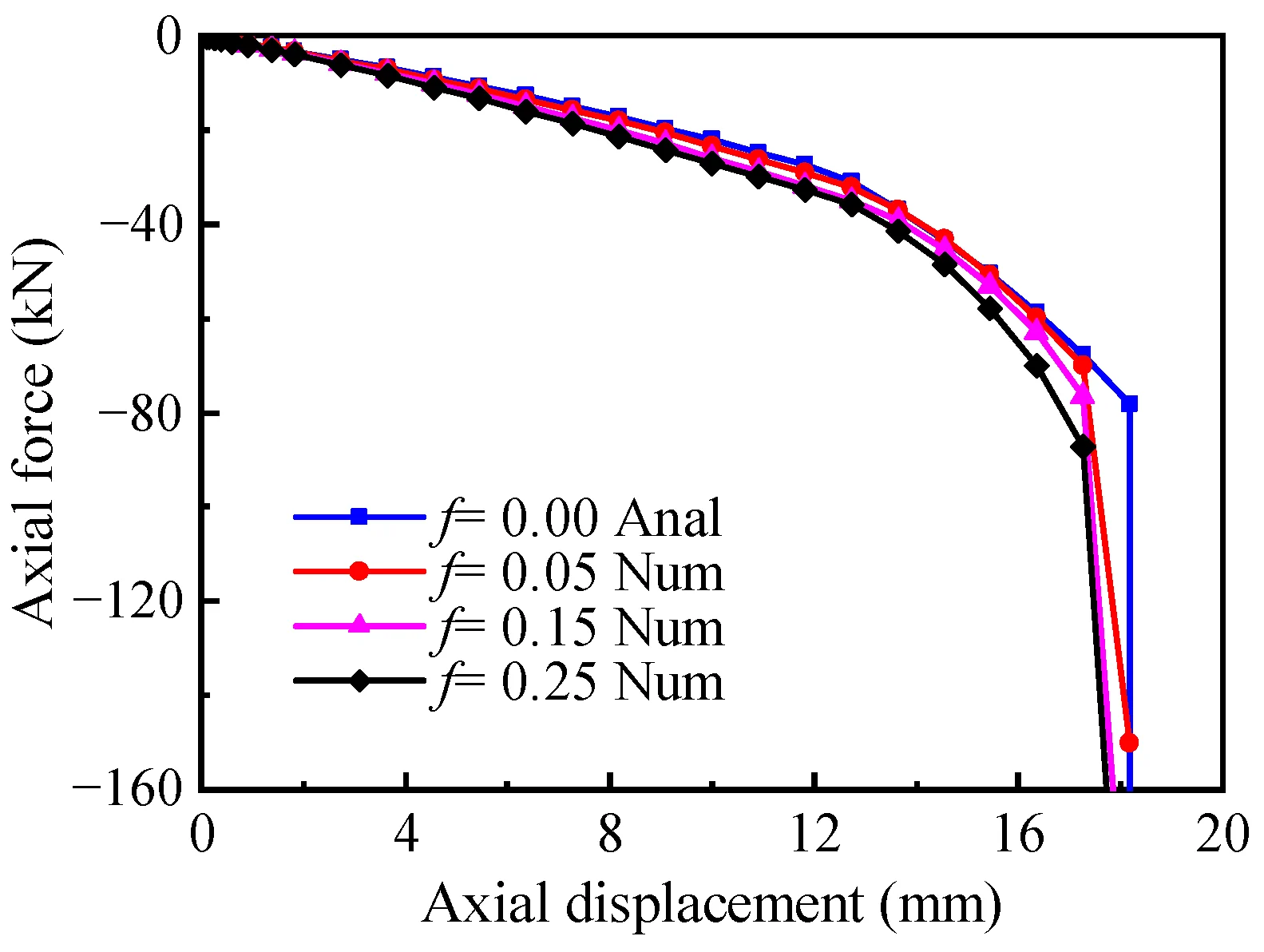
Axial force versus axial displacement for different friction coefficients.

The differences in the deformation process lead to different relationships between axial force and axial displacement. Figure 13 presents the variation in axial force with axial displacement under different friction coefficients. It can be seen from Figure 12 that, due to the effect of friction, the rubber cylinder material cannot completely fill the confined space. Moreover, the larger the friction coefficient, the larger the remaining space. Therefore, when $\lambda_{z}$ approaches $\lambda_{zcr2}$, the axial force shown in Figure 13 increases sharply.

The apparent bulging of the rubber cylinder under frictional conditions should be distinguished from buckling instability. The axial force–displacement curves in Figure 13 increase monotonically for all friction coefficients, with no load plateau or drop characteristic of bifurcation. The mid-height bulging is a side-bulge effect caused by frictional end restraint [40], i.e., a continuous geometric nonlinearity rather than elastic buckling. Moreover, as demonstrated in Section 3.5, the critical compression ratio for the present geometry $\lambda_{zcr2}\approx 0.7353$ lies well above the buckling threshold. Hence, the results of the frictional conditions represent the stable large-deformation path.

**Figure 14:**
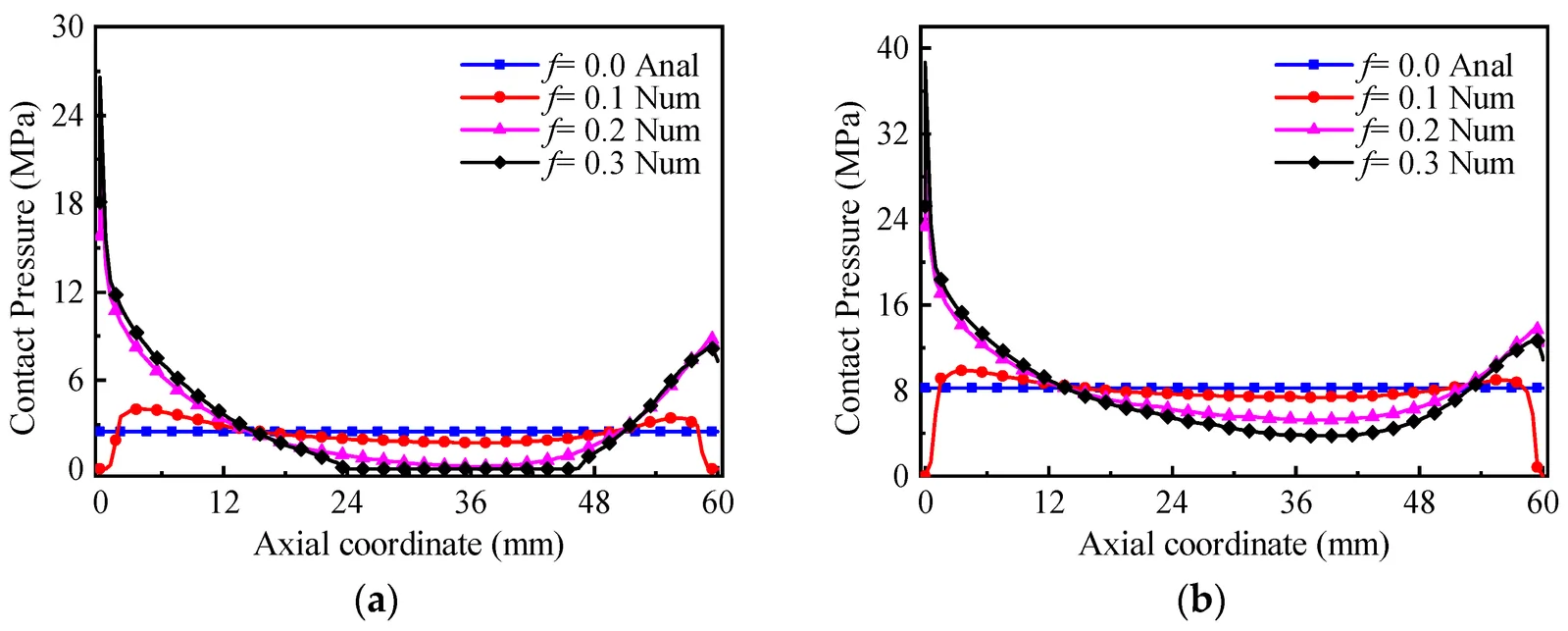
Axial distribution of contact pressure on the inner wall of the rubber cylinder under different axial forces and friction coefficients. (**a**) $F_{z}$ = 100 kN; (**b**) $F_{z}$ = 150 kN.

In engineering practice, engineers are not concerned with the variation process of the axial force, but rather with the axial force required to generate the desired contact pressure. From the previous analysis, it is known that the contact pressure between the inner wall of the rubber cylinder and the mandrel is smaller than that between the outer wall of the rubber cylinder and the casing. Therefore, for different axial forces and friction coefficients, the contact pressure on the inner wall of the rubber cylinder against the mandrel is extracted and compared with the theoretical results, as shown in Figure 14.

It can be seen from the figure that the contact pressure with friction is unevenly distributed along the axial direction, exhibiting a trend of being larger at the ends and smaller in the middle. The larger the friction coefficient, the more pronounced this unevenness. When the friction coefficient is small, the contact pressure is relatively uniform along the axial direction and close to the theoretical result. As the axial force increases, the unevenness of the contact stress becomes more significant.

Table 2 presents the average values of the inner-wall contact pressure under different friction coefficients and axial forces. It can be seen from the table that the average inner-wall contact pressure is very close to the theoretical value. When the axial force is small (100 kN), the theoretical result is smaller than the numerical result, and the difference is relatively large. When the axial force is large (150 kN–250 kN), the difference between the theoretical and numerical results is very small. Therefore, the theoretical result can be regarded as the average value of the contact pressure.

It can be seen in the table that the largest discrepancy between the frictionless theoretical prediction and the frictional FE results occurs at 100 kN. This force level corresponds to the early portion of Stage 3, where the rubber has just contacted the mandrel and the confining effect of the annulus is not yet fully dominant. At this stage, friction-induced non-uniform deformation and end bulging leave unfilled gaps near the ends, which occupy a relatively larger proportion of the available volume. As a result, the frictional perturbation has a proportionally greater influence on the average contact pressure. As the axial force increases to 150–250 kN, the rubber progressively fills the annular space, the confining constraint becomes dominant, and the average contact pressure converges to the frictionless theoretical value. Hence, the 100 kN case represents a local effect confined to the early part of Stage 3, rather than a systematic failure of the analytical model.

**Figure 15:**
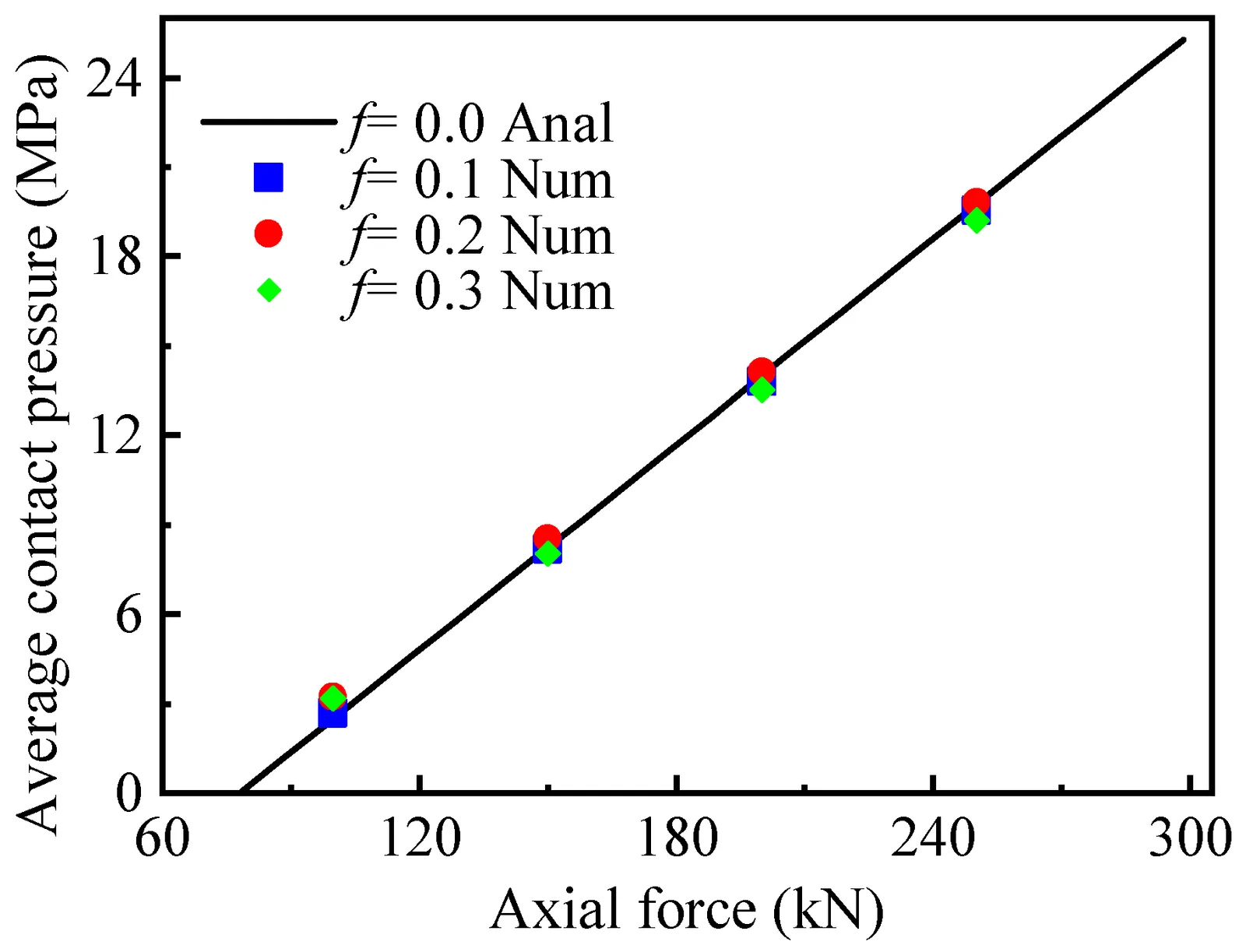
Comparison of theoretical contact pressure with numerical average contact pressure (considering friction coefficients) under different axial forces.

Figure 15 shows the variation in the average contact pressure with the axial force under different friction coefficients. It can be seen from the figure that there is a linear relationship between the average contact pressure and the axial force, which can be described by Equation (34).

The contact stress at the two ends of the rubber cylinder is larger than the average contact stress. This indicates that the liquid pressure applied from one end must first overcome the larger end contact stress before it can penetrate the gap between the rubber cylinder and the mandrel. If the average contact pressure given by the theoretical formula is used as the design basis, the calculated axial force will be conservative. Therefore, the theoretical result has a certain degree of applicability.

It is worth clarifying the scope and limitations of the frictionless analytical solution in light of the frictional results discussed above. While the frictionless assumption cannot capture the axial variation in contact pressure caused by friction-induced non-uniform deformation (as seen in Figure 14, where the frictionless contact pressure is constant along the axis while the frictional cases exhibit distinct variation), it retains practical value for engineering design for two reasons.

First, although the local contact pressure varies significantly along the axial direction under frictional conditions, the axially averaged contact pressure from the frictional FE results agrees reasonably well with the frictionless analytical prediction. For the representative cases with friction coefficient f≤0.3 and axial force $F_{z}\geq 150$ kN, the difference between the averaged frictional contact pressure and the frictionless analytical value is within 3.4%. This indicates that while friction redistributes the contact pressure locally, the overall load-bearing capacity of the rubber cylinder is well captured by the frictionless model.

Second, from a design perspective, the frictionless model provides a conservative estimate. For a given target contact pressure required to achieve sealing, the frictionless solution requires a larger axial force than the frictional model, because friction reduces the net compressive force transmitted to the rubber cylinder. Consequently, a seal designed using the frictionless solution inherently incorporates a safety margin: the predicted axial force is higher than what would actually be needed, ensuring that the sealing requirement is met even under realistic frictional conditions.

Therefore, while the frictionless model should not be used to predict the local contact pressure distribution, it provides a conservative, simple, and physically transparent tool for preliminary engineering design and parameter screening.

## 5. Conclusions

This paper presents a complete theoretical analysis of the axisymmetric compression of an annular rubber cylinder based on the incompressible Mooney–Rivlin hyper-elastic model. The deformation process is divided into three successive stages, and analytical expressions for the stretches, stress fields, and axial force are derived for each stage. The theoretical results are validated against finite element simulations, and their applicability to frictional conditions is examined. The main conclusions are as follows:

(1) A closed-form analytical solution for the axisymmetric compression of an incompressible Mooney–Rivlin rubber cylinder is derived, covering three successive deformation stages: free expansion, casing-constrained deformation, and fully constrained deformation. The solution captures the finite-strain kinematics and yields explicit expressions for the principal stretches, stress components, and axial force.

(2) Under frictionless conditions, the FE results are in excellent agreement with the theoretical results, mutually confirming the consistency of both approaches.

(3) Under frictional conditions, although the rubber cylinder exhibits non-uniform axial deformation and end bulging, the average contact pressure on the rubber–mandrel interface closely matches the theoretical prediction, especially at axial forces above 150 kN. The theoretical result can therefore serve as a conservative estimate of the mean contact pressure for engineering design.

(4) A linear relationship between the average contact pressure and the axial force is identified and quantified by Equation (34). This provides a simple, physically grounded tool for determining the required setting force once the target sealing pressure difference is specified.

It should be noted that the applicability of the frictionless analytical solution to frictional conditions has been verified within the following bounds based on the numerical cases investigated: Mooney–Rivlin incompressible material, coefficient of friction f ≤ 0.3, axial force 150–250 kN, the outer radius and inner radius ratio $1.5\leq R_{o}/R_{i}\leq 2.0$ and the expansion ratio β≤1.2, and axial stretch ratio $\lambda_{z}$ ≥ 0.4477. Extrapolation beyond these ranges requires further investigation.

Furthermore, it should also be acknowledged that the present study compares the analytical results with finite element results, and good agreement is obtained. Direct experimental verification has not been conducted, which is a limitation of the current work. Experimental validation, particularly using indirect measurement techniques such as the sealing differential pressure approach reported by Hu et al. [29], is an important direction for future investigation.

## Figures and Tables

**Table 1:** Geometric parameter table of the packer component.

Structure Name	Geometric Dimension		
	Inner Radius /mm	Outer Radius /mm	Height /mm
Mandrel	29	35.5	200
Rubber cylinder	35.5	56.5	60
Upper and lower ring	35.5	57.5	10
Casing	63.5	69.85	200

**Table 2:** Average contact stress on the inner wall of the rubber cylinder under different axial forces and friction coefficients.

Axial Force /kN	Average Contact Pressure /MPa			
	f=0.0 Anal	f=0.1 Num	f=0.2 Num	f=0.3 Num
100	2.51	2.65	3.21	3.16
150	8.25	8.17	8.53	8.04
200	13.99	13.81	14.12	13.55
250	19.73	19.52	19.82	19.20

## Data Availability

The original contributions presented in the study are included in the article. Further inquiries can be directed to the corresponding author.
